# Safely resuming neglected tropical disease control activities during COVID-19: Perspectives from Nigeria and Guinea

**DOI:** 10.1371/journal.pntd.0009904

**Published:** 2021-12-20

**Authors:** Stephanie McKay, Joy Shu’aibu, Abdourahim Cissé, Albi Knight, Fadhalu Abdullahi, Ahmed Ibrahim, Suzie Madaki, Chantelle Genovezos, Kate McCoy, Philip Downs, Achille Kabore, Helen Adamu, Ibrahim B. Gobir, Michael Chaitkin, Claire J. Standley

**Affiliations:** 1 Independent Consultant, Hinsdale, Illinois, United States of America; 2 Sightsavers, Kaduna, Nigeria; 3 Sightsavers, Conakry, Guinea; 4 Sightsavers, Chippenham, United Kingdom; 5 Sightsavers, Sokoto, Nigeria; 6 Sightsavers, Zamfara, Nigeria; 7 Sightsavers, Durham, North Carolina, United States of America; 8 FHI 360, Washington, DC, United States of America; 9 Georgetown Global Health Nigeria, Georgetown University, Abuja, Nigeria; 10 Independent Consultant, Englewood, Colorado, United States of America; 11 Center for Global Health Science and Security, Georgetown University, Washington, DC, United States of America; University of Hong Kong, HONG KONG

## Abstract

Since its early spread in early 2020, the disease caused by the novel Severe Acute Respiratory Syndrome Coronavirus 2 (SARS-CoV-2) Coronavirus Disease 2019 (COVID-19) has caused mass disruptions to health services. These have included interruptions to programs that aimed to prevent, control, and eliminate neglected tropical diseases (NTDs). In March 2020, the World Health Organization (WHO) released interim guidelines recommending the temporary cessation of mass drug administration (MDA), community-based surveys, and case detection, while encouraging continuation of morbidity management and vector control where possible. Over the course of the following months, national programs and implementing partners contributed to COVID-19 response efforts, while also beginning to plan for resumption of NTD control activities. To understand the challenges, opportunities, and recommendations for maximizing continuity of disease control during public health emergencies, we sought perspectives from Nigeria and Guinea on the process of restarting NTD control efforts during the COVID-19 pandemic. Through semistructured interviews with individuals involved with NTD control at the local and national levels, we identified key themes and common perspectives between the 2 countries, as well as observations that were specific to each. Overall, interviewees stressed the challenges posed by COVID-19 interruptions, particularly with respect to delays to activities and related knock-on impacts, such as drug expiry and prolonged elimination timelines, as well as concerns related to funding. However, respondents in both countries also highlighted the benefits of a formal risk assessment approach, particularly in terms of encouraging information sharing and increasing coordination and advocacy. Recommendations included ensuring greater availability of historical data to allow better monitoring of how future emergencies affect NTD control progress; continuing to use risk assessment approaches in the future; and identifying mechanisms for sharing lessons learned and innovations between countries as a means of advancing postpandemic health systems and disease control capacity strengthening.

## Author summary

Neglected tropical diseases (NTDs) are a group of medically diverse diseases that affect over 1 billion people, predominantly in tropical and subtropical areas, and can cause severe long-term disability and even death. Controlled through mass drug administration (MDA), morbidity management, and/or vector control, NTD programs work closely with communities to identify cases, conduct surveys, and achieve target coverage levels for interventions. The advent of the pandemic of the novel Coronavirus Disease 2019 (COVID-19) in early 2020 led to major disruptions to all facets of life, including health services, with NTD control efforts no exception. Per interim guidelines released by the World Health Organization (WHO) in March 2020, most control activities were halted and only resumed much later in 2020. We sought perspectives from NTD control stakeholders at the subnational and national levels in Guinea and Nigeria regarding the process of planning and executing the resumption of NTD activities to identify challenges, opportunities, and recommendations that might be applicable both to other countries that have experienced disruptions to NTD control efforts during the pandemic and to increase the resilience of NTD programs in the face of future public health emergencies.

## Introduction

In late December 2019, the first cases of the disease caused by the novel Severe Acute Respiratory Syndrome Coronavirus 2 (SARS-CoV-2) Coronavirus Disease 2019 (COVID-19) emerged in Wuhan, China [1]. Since then, the world has seen mass disruptions to daily life, including lockdowns, curfews, economic recessions, and civil unrest. Healthcare systems have been overburdened, and resources in health systems have frequently had to be diverted to deal with the escalating outbreak. The severity of the epidemic was confirmed by January 30, 2020, when the World Health Organization (WHO) declared a public health emergency of international concern, which was rapidly followed in many countries by travel restrictions, border closures, implementation of strict physical distance policies, and other measures aimed at controlling the spread of the virus. In the early months of 2020, and particularly after the epidemic evolved into a pandemic, many health program efforts were either paused or reassessed in order to address the urgent needs related to the pandemic. This included disruptions to programs that aimed to prevent, control, and eliminate neglected tropical diseases (NTDs).

NTDs are a medically diverse set of diseases and disease groups that affect over 1 billion people and can cause severe long-term disability and even death [2]. NTDs are diseases of poverty and are most predominantly found in low- and middle-income countries, especially in tropical and subtropical areas where there may be limited access to safe water and adequate sanitation. Interventions to control NTDs rely on 5 main types of intervention: (1) preventive chemotherapy such as mass drug administration (MDA), usually at community or school level, to address diseases such as schistosomiasis, soil-transmitted helminthiasis, onchocerciasis, trachoma, and lymphatic filariasis (LF); (2) case detection and morbidity management for diseases like leishmaniasis, human African trypanosomiasis, Chagas disease, leprosy, and several others; (3) vector control, as the majority of NTDs are transmitted via an arthropod or molluscan vector; (4) improved water, sanitation, and hygiene (WASH) measures, to address soil-transmitted helminthiasis, schistosomiasis, trachoma, and other NTDs associated with poor water and sanitation; and (5) veterinary public health endeavors, for NTDs with zoonotic reservoirs or veterinary impacts, such as cysticercosis, echinococcosis, and several others [3]. In early 2020, WHO released a new road map for NTDs, setting new targets for control, elimination, and in some cases, eradication, for 2030 [4].

In March 2020, WHO developed and released interim guidelines related to the implementation and planning of NTD control efforts with respect to COVID-19 [5]. This included the temporary cessation of community-based surveys, case detection, and mass treatment interventions; however, morbidity management and vector control were advised to continue to every extent possible. These guidelines were later updated and revised with detailed risk–benefit assessments for NTD program activities. Criteria included potential risk of increased COVID-19 transmission, local public health and social measures, NTD burden in the target population, and expected public health impact of implementing versus skipping or delaying the planned NTD activity (such as mass treatment interventions, active case finding, and population-based surveys), among several other considerations. WHO also developed other guidance that is pertinent for NTD activities, including facility-based operational guidance, WASH guidance, guidance for prevention and control of COVID-19 in special settings, such as in schools and for refugees and migrants, as well as guidance on essential health services. Facility-based guidance included standards for case management prioritization and modifications to standard operating procedures in order to ensure safe practices in the context of the pandemic [6]. Given the impact of COVID-19 on global supply chains, recommendations were made to ensure timely production, shipment, and delivery of NTD medicines, with accommodations. In September 2020, WHO released an overview on the impact of COVID-19 on NTDs [7]. It outlined the key public health consequences of the disruptions to be “an increased burden of NTDs,. . . delays in achieving the public health goals set for relevant NTDs,. . . and reduced collection and analysis of epidemiological data.”

While overall WHO’s African Region has not registered the same devastating numbers of per capita cases and deaths as Europe and the Americas, Guinea and Nigeria have both had sizable COVID-19 outbreaks, with various response protocols put in place to curb the disease (Table 1). Nigeria’s first confirmed case was detected on February 27 2020 (the first case reported in sub-Saharan Africa), and as of September 2021, there have been over 203,000 cases of COVID-19 reported in Nigeria and over 2,660 deaths attributed to the disease.[8] The Nigerian response strategy has been to increase case identification, increase sample collection, and improve contact tracing activities, for example, through establishing over 50 rapid response teams of trained personnel and scaling up PCR-based diagnostic testing [9]. Guinea has reported just over 30,000 coronavirus cases and 374 deaths as of September 2021 [10]. After initially declaring a state of emergency in March 2020, which resulted in a public lockdown, restrictions were gradually lifted. However, a resurgence of cases in February 2021 resulted in renewed measures, including a curfew, mandatory testing prior to leaving Conakry for the interior of the country, and a national mask mandate, with fines for noncompliance [11]. Adding to this challenge, the country declared an Ebola outbreak in February 2021 [12]. Case numbers increased again in July 2021, resulting in a near doubling of the cumulative reported deaths in just 2 months.

**Table 1:** Overview of COVID-19 cases and response protocols in Nigeria and Guinea (as of September 22, 2021).

Country	Nigeria	Guinea
**Cases** ^**1**^	203,081	30,318
**Deaths**	2,666	374
**Response protocol**	• Lockdown implemented during case surges in 2020, schools and borders also closed (quarantine required for travelers); while measures were subsequently eased, many were brought back in May 2021 in response to resurging cases numbers • Over 52 rapid response teams consisting of 352 trained personnel established at state level • PCR diagnostic testing scaled up across the states • Nonpharmaceutical interventions implemented: mask mandate, social distancing, and hand hygiene • Biweekly meeting held with all 36 state epidemiologist and FCT to foster collaboration and coordination	• State of emergency declared in March 2020 • Public lockdown put in place, gradually lifted in mid-2020 • Resurgence of cases in February 2021 resulted in renewed measures, including a curfew, mandatory testing prior to leaving Conakry for the interior of the country, and a national mask mandate (with fines for noncompliance) • Response actions challenged by resurgence of Ebola in February 2021

^1^ Case numbers are based on officially reported confirmed cases, accessed via Our World in Data (https://ourworldindata.org/), and will be lower than actual case numbers.

COVID-19, Coronavirus Disease 2019; FCT, Federal Capital Territory.

Both countries are also endemic for multiple NTDs and have established national control programs, which provide integrated mapping, control, and monitoring and evaluation, supported through funding from various international partners [13]. Five diseases controlled through preventive chemotherapy are endemic in both countries and covered by the respective national NTD control plans: LF, onchocerciasis, schistosomiasis, soil-transmitted helminthiasis, and trachoma. Nigeria’s NTD Master Plan (2015 to 2020) explicitly mentions lymphedema management and hydrocele surgery (hydrocele is a specific type of lymphedema in the scrotum) as necessary interventions for LF elimination. Repeated trachoma infection can lead to trichiasis, a scaring of the eyelid that can lead to blindness; trichiasis surgery is also a key pillar of Nigeria’s trachoma elimination strategy. The Master Plan also covers rabies, leishmaniasis, yaws, dengue, and mycetoma [14]. In Nigeria, the NTD Division within the Federal Ministry of Health (FMOH) provides support and guidance to individual states who are largely responsible for implementation of NTD control activities, in close collaboration with partners. In contrast to Nigeria’s oversight structure for NTDs, Guinea’s control program is centralized within the Ministry of Health. The focus diseases requiring case management in Guinea are leprosy, Buruli ulcer, and human African trypanosomiasis, as well as chronic manifestations of LF and trachoma (such as lymphedema, hydrocele, and trichiasis) [15]. In 2018, the Ministry of Health established a new program dedicated to the control of these NTDs, as well as the 5 combated through preventive chemotherapy.

Overall progress toward NTD targets differs between Nigeria and Guinea. Despite Nigeria’s size, and ongoing challenges with funding availability, logistics, and access to regions affected by conflict, Nigeria was ranked among the top 10 of African countries for its progress toward NTD targets, and it is among the 34 countries that have eliminated at least 1 NTD since 2012 [16]. Guinea, conversely, ranked joint last, although the underlying data largely predated the reorganization of the NTD program, which has substantially reinvigorated control efforts and accelerated progress [17].

NTD control efforts in both Nigeria and Guinea are supported through international assistance, such as the United Kingdom aid-funded Ascend West and Central Africa program [18] and USAID’s Act to End NTDs | West program [19]. Working closely with the respective health ministries, the programs’ implementing partners have continued to provide assistance during the pandemic, for example, through direct support to COVID-19 response efforts, as well as through the development of a risk assessment and mitigation action (RAMA) tool to guide the safe resumption of NTD-related activities [20]. There is a growing recognition of the need to capture the lessons learned from these decision-making and implementation processes, with an emphasis on local perspectives, to maintain progress while the current pandemic continues, and also increase resilience against future threats [21]. To this end, we sought perspectives from Nigeria and Guinea on the process of restarting NTD control efforts during the COVID-19 pandemic, to identify challenges, opportunities, and recommendations for maximizing continuity of disease control during public health emergencies, as well as long-term sustainability and mainstreaming of NTD control.

## Methods

### Ethics statement

The study protocol and questionnaire were submitted to the Georgetown University Institutional Review Board (STUDY00003056) and were assigned a designation “Not Human Research,” precluding the need for further review. However, verbal consent was still obtained from all respondents prior to interviews taking place.

### Country selection and questionnaire deployment

We purposively selected Guinea and Nigeria as the countries from which we would seek perspectives based on the presence of in-country research team members, ongoing collaborations related to NTD control programming, and deployment of the risk assessment and RAMA tool in May 2020, associated with resumption of selected NTD control activities soon thereafter. Although both countries are in West Africa, they vary substantially in geographic area, population size, and health systems structure, among other factors, so we considered them to be promising sources of diverse perspectives regarding the resumption of NTD control efforts.

A questionnaire comprising open-ended interview questions was developed. The questions focused on interruptions to NTD activities due to the pandemic; the process of resuming activities based on WHO guidelines, including challenges and opportunities; and use of NTD resources to support the COVID-19 response. See S1 Text for the full questionnaire.

Input from national, subnational, and local level stakeholders was solicited via in-person (see safety considerations below) or virtual (Zoom or Microsoft Teams) interviews, conducted by employees of Sightsavers or the senior author, using the questionnaire as a template. All in-person interviews took place in accordance with national and local guidelines for preventing SARS-CoV-2 transmission, including use of protective face masks and maintenance of physical distance. Interviews were semistructured, allowing the interviewees to expand on key points and for the interviewers to ask follow-up or clarifying questions as needed. Interviews in Guinea were conducted in French and in English in Nigeria. All interviews were recorded, with the consent of the interviewee, and transcribed in the original language for coding and analysis.

### Data analysis

Interview transcripts were reviewed by 2 authors and coded qualitatively for key themes relating to (1) perceived challenges brought about by the COVID-19 pandemic; (2) perceived opportunities; (3) the use of the RAMA tool (divided into positive and negative/challenging aspects, with reference to its application for restarting NTD control efforts); and (4) recommendations. Key themes were compared across the 2 countries to identify shared perceptions as well as unique observations.

## Results and discussion

**Fig 1:**
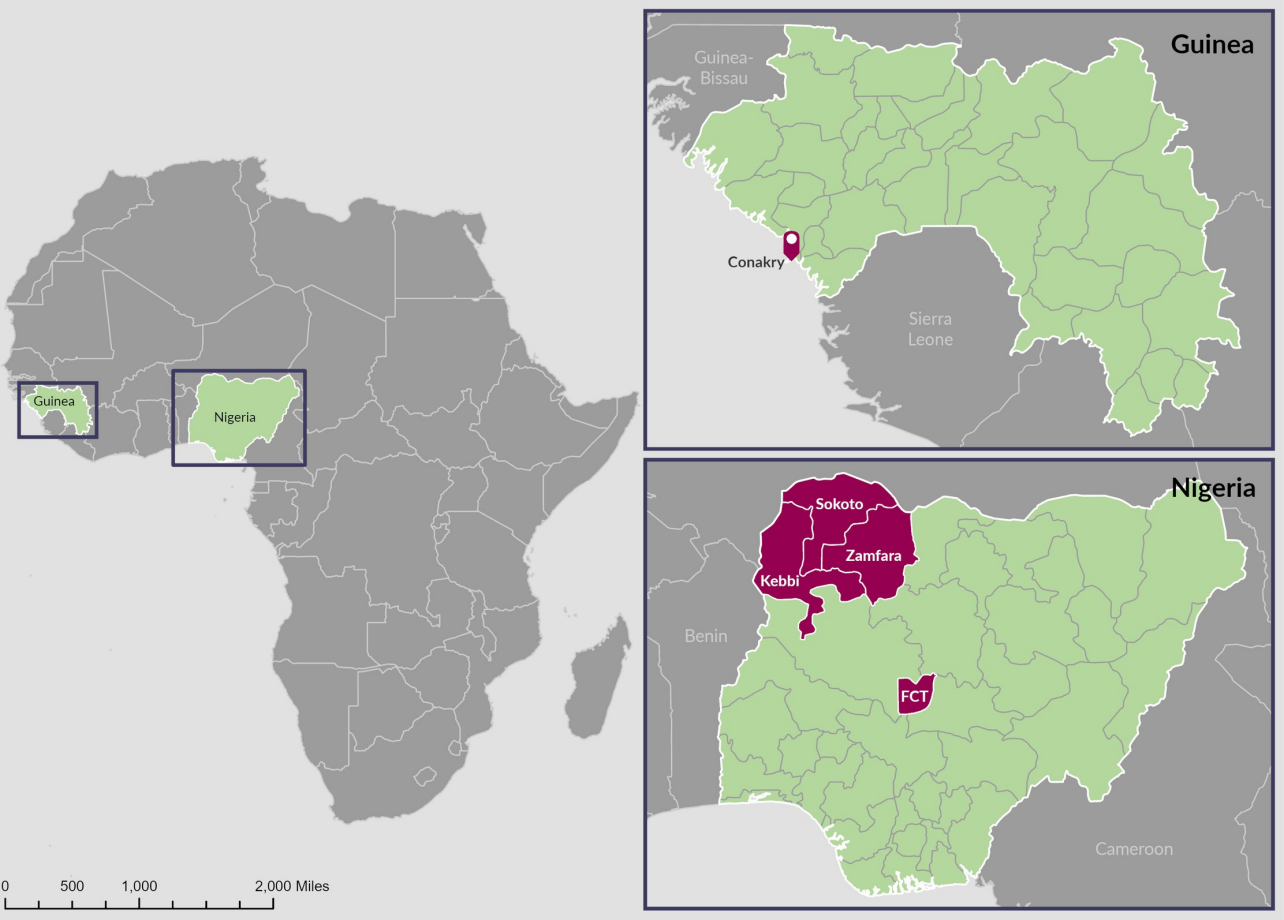
Map showing Guinea and Nigeria and subnational locations of interview respondents. Map layers are sourced from Natural Earth (available from: https://www.naturalearthdata.com/downloads).

In total, we interviewed 3 respondents in Guinea, 2 associated with the national NTD control program (national coordinator and M&E lead) and 1 from Conakry-based Sightsavers country team, and 10 respondents in Nigeria, including NTD control program officials and implementing partners at both federal and state levels (Zamfara, Kebbi, and Sokoto), and 1 community drug distributor (Kebbi) (Fig 1). All but one interview took place in September to October 2020; the final interview took place in February 2021. Thematic responses are summarized in Table 2.

**Table 2:** Challenges, opportunities, and recommendations related to resumption of NTD activities in Nigeria and Guinea.

	Challenges	Opportunities
**Shared perspectives**	• Approval process for activity resumption was slow, due to the extent of engagement with MOH required, as well as the amount of documentation needed as evidence • Concerns over expiry of drugs • Difficulty in directly measuring impact of interruptions on NTD incidence/burden • Could not conduct school-based MDA while schools were closed (or at risk of reclosure) • Concerns that delays would negatively impact elimination schedules • Personnel were concerned about loss of progress and frustrated not to be able to support field activities • Activities were pushed back several months/quarters and took longer to implement	• Use of RAMA facilitated discussion, coordination, and preparation between implementing partners, MOH, and other stakeholders • Discussions provided users with opportunities to identify “low-hanging fruit” as well as solutions for risk mitigation • Coordination has led to greater information and resource sharing between partners and greater ability and confidence among program staff to communicate and advocate
**Nigeria-specific perspectives**	• Disruptions created a backlog of morbidity management cases • Expectation from the FMOH that the tool could be used once for all NTDs, rather than, as intended, for specific interventions or disease programs • Differences in epidemiological situation in different states meant that planning and approvals for resumption of activities occurred at different times • Concerns that COVID-19 economic impacts would have negative repercussions on future availability of donor funding	• National NTD program developed a standard operating procedure for safe resumption of activities for all states to follow or adapt as needed • Webinars and other virtual platforms proved feasible and useful and may continue to be used after COVID-19 (i.e., for savings on travel costs) • Reduced classroom sizes for community trainings have resulted in more effective learning and will be explored as an option post–COVID-19 • Process has required substantial input from communities, which, in turn, has promoted community responsibility and management of NTD control (especially MDA)
**Guinea-specific perspectives**	• Resumption of activities in July 2020 coincided with elections and the rainy season (affecting transportation access); restrictions also had not yet been completely lifted • Coordination of activities, and of overall vision for NTD vision, remains a major challenge • Cost of PPE, posters on transmission prevention, and other COVID-19–related hygiene equipment had not been budgeted for	• Helped to build resilience and trust within the NTD implementation team • NTD program has improved readiness to respond to another pandemic • RAMA tool helped mitigate fears over transmission and potential risks of restarting programming, at all levels (managerial, operational, and field teams) • Adapted MDA included provision of COVID-19 information to households, which increases community buy-in for both NTD control and COVID-19 response efforts • An NTD/COVID-19 supervision tool was developed and introduced during MDA; its success means it has since been rolled out via implementing partners to other West African countries
**Recommendations**	• There is a need to ensure greater access and availability to historic data on NTD prevalence and distribution, for example, through creation of national or regional databases, so as to better understand the impact of disruptions like COVID-19 on existing patterns and trends • A learning tool should be developed to complement initiatives like RAMA and ensure rapid application of risk assessment processes in the future • Key partners should continue to be sensitized on the RAMA tool; risk assessment is a helpful process for identifying barriers as well as opportunities for successful program implementation and so could be applied usefully in the future (and the pandemic is not yet over) • Lessons learned from COVID-19, as well as innovations developed to safely resume activities, should be shared between countries, as well as with national COVID-19 taskforces; some innovations may be beneficial to continue postpandemic	

COVID-19, Coronavirus Disease 2019; FMOH, Federal Ministry of Health; MDA, mass drug administration; MOH, Ministry of Health; NTD, neglected tropical disease; PPE, personal protective equipment; RAMA, risk assessment and mitigation action.

### Opportunities and successes

Respondents cited a number of different areas where they perceived opportunities for improved NTD control as a result of the pandemic or where the adaptations required to activities may have lasting benefits, including through improved advocacy and coordination; sharing of tools, resources and information; sustainability and integration; preparedness; and innovation. The RAMA tool was also specifically cited by respondents in both countries as a successful intervention allowing for safe and responsible resumption of NTD control activities.

Across both countries, most respondents described how the need to identify risks, develop mitigation approaches, and gain consensus across different stakeholder groups led to increased coordination between partners, which, in turn, has facilitated sharing of information, tools, and other resources. For example, in Guinea, during the development of the NTD contingency plan, derived from application of the RAMA tool, the NTD control program team had to be very proactive with engagement and advocacy across multiple levels of the government, which has now resulted in much greater awareness of NTD control efforts among parliamentarians, civil society, and other stakeholders who previously had been less closely involved in NTD decision-making, although there may still be opportunities to enhance the spread and quality of messaging specific to COVID-19 mitigation and NTDs. Other examples of how communication and coordination has been enhanced included the widespread use of webinars and other virtual platforms, particularly cited by Nigerian respondents, as a very useful way to share information, and which may continue to be used as a means of reducing programmatic travel costs.

Overall, the RAMA tool was highlighted by most respondents as very useful in allowing for the resumption of NTD activities, particularly in terms of having support for decision-making and providing reassurance that activities were safe to resume. In addition, the identification of risks allowed opportunities for solutions to be presented and discussed collaboratively. Indeed, the benefits of the tool went beyond the risk assessment process itself. Respondents in both Guinea and Nigeria noted that the use of the tool facilitated greater discussion and collaboration between NTD program officers, implementing partners, as well as the MOH. In some cases, this led to a slow approval process, but it also helped gain support from all necessary parties to resume activities. The process of utilizing the tool was also cited by Guinean respondents as helping to build resilience and motivation within the NTD control program team and has contributed to building the communication skills of the program as well as individuals within it. Knowledge that activities had undergone a risk assessment process was also seen as a benefit in terms of enhancing community trust and engagement in both countries. The tool itself was described as being intuitive to use and clear, although the extensive documentation required as evidence was another source of delay. Nigerian respondents noted that the FMOH had thought the tool could be utilized once across all NTD programs, whereas in fact it had been developed with the intent to use sequentially on different disease efforts, to assess the risk across each set of discrete activities.

Respondents, primarily in Nigeria, highlighted a number of ways in which NTD control activities have been integrated into the COVID-19 response, which may, in turn, provide opportunities for longer-term health systems mainstreaming and sustainability, in line with WHO 2021 to 2030 Road Map. For example, in Sokoto state, the NTD control program and implementing partners have contributed to hand hygiene initiatives, including donation of soap and other materials, which, while designed to target COVID-19, will likely have secondary benefits with respect to reducing transmission of NTDs and other infectious diseases. While NTD programs were already working to cross-collaborate with veterinary and WASH programs (among others), there may be a renewed global interest after the pandemic in such cross-cutting interventions and integrated approaches. Similarly, in Kebbi state, the NTD control program vehicle, when not required to support NTD efforts, has been used for COVID-19 response activities. In some states, all health workers have been trained to identify and refer suspected cases of COVID-19. In Guinea, the resumption of MDA has included sharing of information related to COVID-19 transmission and risk reduction to participating households. These sorts of “hybrid” programmatic approaches may serve to add resilience to NTD control activities, as well as provide a blueprint for other integration opportunities with other health services [22]. Moreover, respondents in both countries noted that these additional efforts to engage with communities have served to enhance community buy-in of NTD programs and promote community responsibility for disease control.

Two final key areas of opportunity relate to improved preparedness and the encouragement of innovations to address control challenges, which can be leveraged to improve programming in the future. For example, in Guinea, respondents reported that they feel better prepared for future pandemics or epidemics, based on their experiences during COVID-19, and are confident that disruptions can be minimized through these lessons learned. The utility of the risk assessment tool prompted respondents to suggest that similar tools could be developed to ease the impact of other types of future disruptions as well, for example, due to extreme weather events or political instability. The challenges of the pandemic also spurred innovative thinking with regard to design and deployment of NTD control activities. For example, in Guinea, a COVID-19/NTD supervision tool was introduced during MDA, using an electronic platform to capture data. The tool was successfully adapted to other West African countries and could provide a useful resource in the future. In both countries, NTD control programs explored novel, contactless ways of performing key activities in an effort to reduce COVID-19 transmission risk. Contactless height poles were used for drug dosing, for example, and trays or spoons were used to distribute drugs. Finally, some of the changes forced by the risk mitigation measures may actually have benefit in their own right. For example, in Zamfara state in Nigeria, respondents noted that community drug distributor training was much more effective during the pandemic than in previous years, which they attributed to smaller class sizes. Prior to the pandemic, 60 to 80 individuals would be trained at once, whereas the distancing requirements during COVID-19 meant only 20 individuals could be trained per session in 2020. Instructors noted that trainees paid closer attention to the material, and class control was more easily managed, and they are hoping to be able to sustain these smaller class sizes in the future; whether this signals a more cost-effective approach is worth further investigation.

### Challenges

This pandemic comes at a time when great strides have been made on NTDs. Since 2015, 1 billion people have been treated for at least 1 NTD, including 556 million for LF and 114 million for onchocerciasis. Guinea worm disease remains on the verge of eradication, with only 53 cases globally in 2019 [23]. COVID-19 also immediately preceded the launch of the new NTD 2021 to 2030 Road Map, meant to be a guiding document for the global response to NTDs for the next decade.

With that said, the pandemic poses an obvious and unprecedented challenge to the plans of many NTD programs [21]. Modeling has shown that many programs may face a risk of case resurgence, especially in high-transmission areas, unless interventions are reintroduced [24]. Impact will likely vary across disease programs, based on factors such as rate of transmission of particular NTDs and level of baseline endemicity [25]. The complete cessation of MDA—and associated shifts in programmatic timetables once activities resumed—had knock-on impacts related to the availability of drugs—for example, the risk of drug expiry was listed as a challenge in both countries, and this affected the prioritization of some NTD programs in Nigeria—with some elimination timelines and logistics. For example, in Guinea, resumption of activities coincided with the rainy season and elections. While respondents noted that the threat of the upcoming elections provided useful time pressure to incentivize getting activities underway, and in the end did not cause additional disruptions, the poor road conditions associated with the rainy season impeded implementation progress. Meanwhile, ongoing COVID-19–related restrictions in both countries, such as school closures, required a further shift in implementation approach to delivery at the household level. In Nigeria, community health workers reported challenges in vertical program implementation as healthcare workers were shifted to other program activities related to COVID-19. Furthermore, interruptions to routine health services created a substantial backlog of morbidity management cases in Nigeria. Finally, the disruptions negatively impacted team morale, with personnel frustrated at not being able to support field activities, while also anxious about the risk of COVID-19 for themselves and their communities. Respondents did not describe competition for health workers, personal protective equipment (PPE), and medical supplies as having impacted the ability of NTD programs to operate [26], but interviews in Guinea highlighted that the need to procure and use PPE when implementing activities, along with other COVID-19–related costs like prevention posters, had not been budgeted for in the previous year’s planning, causing difficulties with ensuring availability of sufficient operational funds.

Although it did not feature extensively in our interviews, there is also the question of whether the short-term increase in health expenditure to support COVID-19 response efforts, and pandemic preparedness more broadly, will translate into larger domestic health budgets, including for NTDs, in 2021 and beyond. In low- and lower-middle income countries, increases in public spending on health are driven most by economic growth and expanded overall public spending, both of which are suffering substantially due to the pandemic [27]. One national level respondent in Nigeria noted their concern that the pandemic’s economic harms could both cause or exacerbate domestic fiscal constraints and erode donors’ enthusiasm to spend on nonemergent global health needs. The latter seems to have been borne out by recent announcements regarding UK cuts to development assistance generally and to NTD programs in particular [28]. In any scenario, the short- and long-term effects of the pandemic on funding for health and NTDs will depend on a range of issues, including the share of spending financed from domestic versus external sources, broader fiscal trends, and the ability of NTD program officials and their allies to mobilize and sustain political support both domestically and among key development partners. Taken together, these challenges suggest that there may need to be a reconsideration of what “sustainability” and mainstreaming of NTDs will look like as health systems continue to manage and ultimately overcome COVID-19.

### Recommendations

The perspectives of respondents across both Nigeria and Guinea revealed a number of key recommendations for future action to improve resilience and sustainability of NTD control, particularly in the face of future disruptions.

First of all, there is a need to improve data availability and analysis on disease prevalence and burden, as well as access to historical data, in order to better anticipate and plan for potential disruptions to activities. Modeling from WHO has shown that delays to MDA could forestall achievement of the 2030 targets by 1 to 3 years, depending on the disease and length of MDA delay. Modeling was also done of the impact of delays on Gambiense human African trypanosomiasis, LF, onchocerciasis, schistosomiasis, soil-transmitted helminthiases, trachoma, and visceral leishmaniasis in the Indian subcontinent. Models on the impact of treatment delays can give governments and program managers an improved understanding of these impacts and thus inform prioritization for future intervention and enhanced preparedness. Continued forecasting will be a necessary tool to mitigate the impact of COVID-19 on future NTD programming [29]. Critically, ownership of these data must rest with the national NTD control programs and the Ministry of Health [30]. One option is for national governments to mandate that researchers and implementing partners focused on NTDs in endemic countries commit to providing the outcomes of their investigations and program evaluations to the national NTD control program. Donors and partners can also support improved electronic capture of data, and capacity strengthening assistance in database management, to ensure national ownership of these databases, as evidenced by the successful deployment of the COVID-19/NTD management tool in Guinea, and its rollout to additional countries in West Africa. Others have noted that the uptake of digital tools can also help with micro planning of MDA, which are used to help track targets for immunization campaigns (including polio) [29]. These tools can help improve the large-scale tracking of program targets, as well as identifying gaps in program outreach or any areas where coverage has lapsed.

The examples of Guinea and Nigeria highlight that NTD programs have been able to resume safely even while the COVID-19 pandemic has been ongoing, but such efforts are best carried out through a very deliberative and consultative process. While this may slow down implementation, it allows for greater buy-in at all levels and provides critical reassurance to program officials, project staff, and communities alike. Tools like RAMA can facilitate this consultative process and also identify key areas where mitigation is most needed; importantly, they can also build long-term support for NTD control, through engaging nontraditional stakeholders and expanding the reach of NTD advocacy. Development of a learning tool to accompany RAMA or similar risk assessment tools could help to fast-track their deployment and successful application in future pandemics or disruptions. Others have also proposed that reintroduction of programs can be an opportunity to accelerate progress toward 2030 goals, such as through biannual MDA for trachoma and onchocerciasis, more effective drug combinations (including triple drug therapy as an option in areas without onchocerciasis), or expansion of programs in high-transmission settings [24,25].

As seen in both Guinea and Nigeria, albeit in the limited locations included in this study, NTD control programs can be successfully leveraged for the COVID-19 response and can enhance their own preparedness to future interruptions in so doing. Similar outcomes have also been observed in other countries in West and Central Africa [30]. More generally, these approaches provide opportunities for identifying further areas for activity or programmatic integration, either within the health system or through allied services such as WASH. Risk communication may be an area particularly ripe for closer integration between NTD programs and pandemic response. Indeed, the success of community engagement and health promotion enjoyed by NTD control programs while resuming activities during the pandemic suggests other key areas in which the programs can be leveraged to support the response, such as vaccination campaigns.

In particular, the approaches that NTD programs take for MDA can be applied readily to COVID-19 vaccine rollout [31]. Strategies like social mobilization, transparent communication, and community leader–based advocacy help to improve the public’s trust in health interventions. Health programs should seek to eliminate as many barriers as possible, which MDAs have achieved in creative ways, including bringing medications to people’s homes and schools, even in hard to reach areas. There may be further benefits to aligning COVID-19 vaccination campaigns with NTD MDA; schistosomiasis infection, for example, has been shown to reduce the efficacy of other vaccinations, suggesting that adults in endemic regions or higher-risk occupations (i.e., fishermen) should be offered praziquantel alongside the COVID-19 vaccine [32]. Overall, there remain substantial opportunities to think out of the box in terms of NTD integration and “hybrid” approaches to disease control, including future pandemic preparedness and response efforts [22,33].

### Limitations

There were several limitations to this study. This evaluation involved interviews with a small number of stakeholders in both countries, from a limited set of geographical areas. As such, a broader, multisectoral effort would need to be made to get a fuller picture of COVID-19’s impact on these countries’ NTD programs. Due to the localized nature of the interviews, the findings from this study cannot necessarily be externalized to other contexts.

Another limitation is due to the fact that the pandemic is still ongoing and, in many ways, worsening. While multiple vaccines are being rolled out widely across high-income countries, many countries in Africa are not expected to achieve sufficient vaccination coverage for several years, with full coverage on the continent unlikely before 2024 [34]. However, rollout of COVID-19 vaccines began in several African countries at the start of 2021. Nigeria received its first batch of vaccines on March 2, 2021 through the COVAX facility, which included 3.92 million doses of the Oxford/AstraZeneca vaccine [35]. Health workers are the first targeted for vaccination. The Russian government has donated doses of the Sputnik V vaccine to Guinea, starting with President Conde in January 2021 [36], and China has also pledged to donate 200,000 doses to the country [37]. While these are clearly positive developments, the emergence of the highly transmissible Delta variant in early 2021 led to devastating resurgence of cases in South Asia, followed by a near global increase in transmission and case numbers, even in countries with high vaccine coverage. The African region experienced its highest weekly number of fatalities from COVID-19 at the beginning of August 2021, highlighting the continued threat posed by COVID-19 to societal, economic, and health systems recovery [38]. To this end, NTD programs must consider risk assessment as an ongoing process and leverage the communication, advocacy, and coordination skills enhanced over the past year to continue working closely with governmental and other key stakeholders to ensure safe continuity of operations.

The perspectives presented here of course also represent a snapshot in time, with interviews taking place in late 2020 and early 2021. The longer-term impacts of COVID-19 on NTD control programs, as well as the trajectory of the pandemic itself, remain to be seen. Both Nigeria and Guinea have NTD programs that are strongly supported by external funding and implementing partners, so in both these countries, there may be more immediate impacts observed in the next fiscal year. Further to this point, as the majority of interviews were conducted by Sightsavers staff, respondents may have been reluctant, consciously or unconsciously, of providing negative feedback on the use and application of the RAMA tools, knowing that these had been developed by the Ascend Partners.

The 2021 Ebola virus disease outbreak in Guinea also added a layer of complexity to both the COVID-19 response and ongoing efforts to provide NTD services to the population. With that being said, the country has experience with handling the disease and, as of May 2021, was able to rapidly contain the outbreak through deployment of response teams and rapid vaccination rollout [39]. If no further cases are identified, the outbreak will be declared over on June 19. Similarly, Nigeria’s surveillance system has remained relatively robust despite the added pressures from COVID-19, with ongoing surveillance for Lassa fever, yellow fever, measles, and other priority diseases, although security and economic challenges continue to pose difficulties, especially in northern states. This demonstrates that despite concerns over limited human and material resources, health systems in Guinea and Nigeria may be fairly resilient in the face of emerging threats, which could prove important with respect to future mainstreaming and sustainability of NTD and other endemic disease control programs.

## Supporting information

S1 Text Questionnaire for semistructured interviews with key informant respondents. (DOCX)
