# Supplementary material for: Safely resuming neglected tropical disease control activities during COVID-19: Perspectives from Nigeria and Guinea
Source: PLoS Negl Trop Dis. 2021 Dec 20;15(12):e0009904. doi: 10.1371/journal.pntd.0009904 (PMC8687572; doi:10.1371/journal.pntd.0009904)
Supplement: S1 Text — (DOCX) [file pntd.0009904.s001.docx]

**S1 Text**

**Case study guiding questions**: Country experiences with implementing NTD control during the COVID-19 pandemic

- During the COVID-19 pandemic, did your country’s NTD program partially or fully cease activities?
  - If partially: Which activities were able to continue? How were these programs assessed? Were any adaptations made to implementation to minimize risk of COVID-19? What guidance, if any, was followed or implemented to decide how activities should proceed in light of COVID-19?
  - If either partially or fully: Has your country estimated the impact of the reduction in control efforts on the prevalence or morbidity associated with NTDs? Will these data be used to guide targeting of future control/elimination efforts?
- Is any part of the NTD control program (control program staff, infrastructure, community personnel, supplies, etc.) being used to support COVID-19 response activities?
  - If yes: What sort of activities are being supported, and by which personnel? Do these personnel normally work full-time for the NTD control program? Will their new activities/responsibilities for COVID-19 continue even when NTD control efforts resume?
- After the release of the new WHO guidelines, has your country taken steps to restart any NTD control/elimination activities?
  - If yes: What is the process for determining which activities should resume first? What steps are being taken to adapt implementation efforts to minimize the risk of COVID-19? Are you using any risk assessment processes to help guide implementation decisions?
  - If no: What factors are preventing NTD control programs from restarting?
- What are the specific challenges associated with restarting NTD control activities while the COVID-19 pandemic is still on-going?
- Have you seen any evidence of co-morbidities relating to NTDs and COVID-19?
- Have you seen any evidence related to excess NTD morbidity / mortality due to changes in implementation of control programming related to the COVID-19 pandemic?
- How has COVID-19 affected funding streams for NTD programs? (For example, consider drug supply chains, staffing, etc.)
  - If your NTD program is supported by MOH/national government funding: Has the budget for NTDs from the MOH been cut or redirected? Have you received any funds from the MOH/national government, and if yes, for which activities/programs? If you have prepared your budget for next year, have there been any changes related to NTD control/elimination activities?
- Are there examples of potential successes for integration or mainstreaming of NTD control program that have come about as a result of the COVID-19 pandemic?
  - If yes: Do you see these changes as one-off occurrences, to manage the impact of the pandemic, or is there a sense that these could be opportunities for longer-term integration or innovation?
- Do you think that there might be greater openness to innovation or experimentation in the way community services are organized, to account for the pandemic (or future disruptions)?
